# Subcellular localization of C-term-oleosin fused to β-casein reveals unexpected cytoplasmic accumulation in vacuole-targeted arabidopsis seeds

**DOI:** 10.3389/fpls.2026.1872014

**Published:** 2026-06-30

**Authors:** Almog Ozeri, Mai Shamir, Miron Abramson, Barak Cohen, Amir Rudich, Nitsan Lugassi, Oded Shoseyov

**Affiliations:** 1 Robert H. Smith Faculty of Agriculture, Food and Environment - The Hebrew University of Jerusalem, Rehovot, Israel; 2 Miruku Ltd., Rehovot, Israel; 3 GeneNeer Ltd., Ness Ziona, Israel

**Keywords:** arabidopsis seeds, molecular farming, oil bodies, oleosin, proteolysis, recombinant protein accumulation, subcellular compartmentation, vacuole

## Abstract

Casein is a high-quality protein source containing all amino acids, which are vital for human nutrition, and play a vital role in granting the texture and mouthfeel of dairy products. Plant-seed production may be an efficient strategy for alternative protein production due to its ability to produce complex proteins, perform post-translational modifications, and be resource-efficient. Targeting recombinant proteins to specific subcellular compartments plays a crucial role in ensuring proper folding, stability, and accumulation. To determine the most suitable subcellular compartment for accumulation of bovine β-Casein fused to plant C-terminal Oleosin (CTO-Cas) different signal peptides were tested, directing the chimeric protein to endoplasmic-reticulum, vacuole or to the chloroplast. These gene constructs were transformed into Arabidopsis using Agrobacterium-mediated transformation by the floral dip technique. CTO-Cas was successfully detected in transformed seeds which were targeted to the vacuole, measured at 1.26% of the Total Soluble Protein (TSP). Subcellular compartmentation of the vacuole-targeted chimeric protein was determined by Transmitting Electron Microscopy (TEM) using gold-immuno-labeling. CTO-Cas was detected in spherical bodies, which are not vacuoles. Major morphological changes were observed in the transgenic seed cells in comparison to WT. Large oil bodies dominated WT seed cells, while seeds expressing CTO-Cas targeted to the vacuole had fewer large oil bodies. Instead, they exhibited numerous small oil bodies alongside dense sub-cellular aggregates only observed in the transgenic seeds. These aggregates were positively gold-immunolabeled with anti-Casein antibodies as well as anti-oil body associated protein antibodies, suggesting the formation of recombinant CTO-Cas aggregates tightly associated with small oil bodies.

## Introduction

By 2050, the global population is projected to reach 9.6 billion, leading to an increased food consumption compared to 2010 baselines, and more specifically, an estimated 58% increase in dairy protein consumption. Foods derived from animals account for a significant portion of the energy and protein in diets worldwide. Livestock provides 34% of the protein consumed globally along with essential amino acids, energy, and nutrients (such as iron, zinc, and vitamin B12), which can be difficult to obtain from a plant-based diet alone (Smith et al., 2024). There are, however, serious consequences associated with the production of animal-based proteins; For instance, recent conservative estimations by the Food and Agriculture Organization (FAO) of the United Nations suggest livestock accounts for about 14.5% of global greenhouse gas emissions (Tian et al., 2024) Moreover, nitrous oxide emissions from fertilizers used to grow feed for livestock are further contribute to greenhouse gas emissions, surpassing those caused by fertilizer application on food crops. Finally, manure and effluent waste produced by dairy farming contribute to significant eutrophication, where nutrient runoff from livestock operations pollutes potable water resources in both nearby and distant communities (Gaillac and Marbach, 2021). Consequently, a more sustainable yet equally nutritious non-animal substitute resource is required. While there is a growing adoption trend of alternative-sourced options to traditional dairy products, there is still a significant gap in achieving a non-animal alternative that truly mimics the taste, smell, texture, digestibility, functionality, and nutritional value of conventional dairy.

Bovine milk is an aqueous-based emulsion containing approximately 12.7% total solids, with proteins accounting for roughly 3.2% of the total composition. These proteins are categorized into two primary groups: the soluble whey proteins and the insoluble casein fraction (Fox et al., 2015) Casein, comprising about 80% of milk’s protein content, exists mostly in micelles - complex spherical structures stabilized by calcium ions and hydrophobic interactions (Rebouillat et al., 2015). Considered as a complete and slow-digesting phosphoprotein’s, Casein’s provides a gradual release of all essential amino acids, maintaining a longer feeling of satiety. Moreover, Casein’s unique structure and composition make it an excellent ingredient for various dairy food applications. Casein is essential for creating a stable emulsion in milk, its derivatives and alternatives, providing the characteristic texture and mouthfeel of dairy products and enabling the melting and stretching properties of cheese (Runthala et al., 2023). Four Casein protein families (κ-, β-, αS1-, and αS2-Caseins) have evolved in different mammalian species to maintain specialized roles in milk. Of this protein family, β-Casein, considered a major protein in milk composition, comprises about 30-35% of total dairy proteins. Its amphiphilic nature, with a hydrophilic C-terminal and a hydrophobic N-terminal, is key to its function in self-assembling micellar structures (H. M. Farrell et al., 2002).

Producing recombinant dairy proteins such as β-Casein, opens new opportunities for producing highly specific dairy proteins that can overcome some of the challenges associated with conventional dairy farming. Plant Molecular Farming (PMF) has emerged as a promising technology, leveraging plants as cost-effective and scalable bioreactors for the production of valuable recombinant proteins. This versatile platform is a viable tool for developing both functional foods and affordable biopharmaceuticals, presenting a sustainable alternative to traditional production systems (Shanmugaraj et al., 2020; Long et al., 2022). A key advantage is the plant’s ability to produce complex proteins and perform the necessary post-translational modifications, ensuring a high-quality product (Stein et al., 2009). The inherent simplicity of agricultural growth, which requires only water, minerals, and sunlight, coupled with the superior storage and distribution properties of plant-derived products, makes this approach particularly attractive. While PMF holds immense promise, a critical evaluation of its achievements also underscores significant future challenges, including regulatory frameworks, public acceptance, and the need for efficient downstream processing (Schillberg and Finnern, 2021).

Primary challenge in producing animal proteins in plants is achieving high yields, this is influenced by factors such as gene transcription, translation efficiency, protein stability, and the plant species or tissue of expression (Schillberg and Spiegel, 2022; Tusé et al., 2024).

Despite these challenges, the strategic application of PMF is crucial for enhancing global food security by providing a robust and affordable means to produce nutrient-rich functional foods on a large scale.

Plant molecular farming has proven itself in the production of highly valuable proteins, for example in the pharmaceutical industry (Ma et al., 2003; Morandini et al., 2011). For instance, studies have successfully demonstrated the production of complex proteins such as human pro-insulin in Camelina sativa seeds, which exhibited anti-diabetic efficacy in animal models, and the molecular pharming of hEGF fused to oleosin in transgenic Arabidopsis (Bhoria et al., 2025; Qiang et al., 2020). Efforts to express recombinant dairy proteins in plants have shown limited but promising results. Of note to this paper, early studies focused on expressing caseins in various crops have been reported. For instance, the accumulation of human β-Casein in Solanum tuberosum (potato) was reported at a concentration of 0.01% of the total soluble protein (TSP) (Chong et al., 1997). Similarly, Maughan et al. (1999) achieved a slightly higher expression of bovine β-Casein in Glycine max (Soybean), with a range of 0.1-0.4% of TSP (Sovyanhadi et al. (2001) also documented the expression of β-Casein in Solanum lycopersicum (Tomato) at 0.01-0.05% of TSP. These studies, while demonstrating the feasibility of using plant systems for producing dairy proteins, highlight the challenge of achieving high expression levels.

Post Translational Modifications (PTMs) of β-Casein have been employed to improve its solubility, stability and bioavailability, which are crucial for the nutritional and physiological functions of dairy products. Specifically, phosphorylation of β-Casein enhances its solubility and prevents premature aggregation, ensuring proper micelle formation and nutrient stabilization (Antuma et al., 2023; Mora Vásquez et al., 2025). β-casein contains multiple phosphorylation sites, predominantly on serine residues. This post-translational modification is critical for regulating protein function and is carried out by mammalian casein kinases, such as the FAM20C Golgi associated secretory pathway kinase (Palma-Lara et al., 2021; Chen et al., 2021). This process modifies the structure and function of secreted caseins, enhancing their ability to bind minerals like calcium and interact with other extracellular proteins. However, the reliance on ATP for phosphorylation in plant-based expression systems is energetically demanding, placing a significant metabolic burden on the host plant (Antuma et al., 2023). To overcome the need for mammalian Casein kinase expression in host plants, an alternative strategy called “Phosphomimicking” has been developed. This approach replaces phosphorylatable serine residues in β-Casein with negatively charged amino acids, such as glutamate or aspartate, mimicking phosphorylation. The resulting protein retains key functional properties, including micelle stability and calcium-binding affinity, without requiring enzymatic phosphorylation. Phosphomimicking not only streamlines downstream processing of recombinant β-Casein but also reduces production costs and enhances scalability in plant expression systems (Acevedo Fani and Abramson, 2023).

Since dairy milk is an emulsion of protein and lipid droplets in water, oilseed crops with optimal protein-to-oil ratios might facilitate the development of plant-based dairy products with desirable emulsification properties. In addition, directing recombinant proteins to seeds by using seed specific promoter has advantages (Dean and Finer, 2023). During development, endosperm cells naturally produce and store proteins and other nutrients (oils, carbohydrates, etc.) to nurture the developing plantlet after germination (Li and Berger, 2012). Thus, the target protein expressed in the seed finds a cellular environment that favors protein accumulation. For example, the seed storage protein Cruciferin (UniProt: Q96318) accumulates within the protein storage vacuole (PSV), and responsible to 60% of the seed’s overall protein content, representing around one-quarter of a seed’s dry mass (Nguyen et al., 2015; Rolletschek et al., 2020).

Arabidopsis (A. Thaliana), a model plant widely used in research, contains significant amounts of oil in its dry seeds; typically ranging from 34.6% to 46.0%. Oil bodies, also known as oleosomes, are specialized organelles found in plant seeds that store lipids. Each spherical oil body has a diameter of about 0.5–2.0 μm and consists of a triacylglycerol matrix surrounded by a layer of phospholipids embedded with proteins called oleosins (Huang, 1994). These proteins play crucial roles in maintaining oil body structure, stability, and function during seed development, maturation, and germination. In the context of recombinant protein expression, studies revealed that by fusing oil-soluble oleosin motifs to other proteins may facilitate their extraction, enabling easy and efficient purification via flotation and centrifugation (Ling, 2007; Bhatla et al., 2010; Şen et al., 2024). According to pending patent P10199WO, β-casein fused to the C-terminal oleosin was expressed in E. Coli., the resulting recombinant protein demonstrated efficient binding to plant oil bodies.

While oleosins may be introduced to facilitate oil-binding, plant cells also contain specialized, membrane-bound organelles that separate and optimize distinct biochemical processes (Solymosi and Schoefs, 2019). Short signal peptides, present at the N-terminus of the most newly synthesized proteins, are wildly employed by plants and help direct these proteins to their destined secretory pathways (Kaushik et al., 2022). Targeting recombinant proteins to specific subcellular compartments plays a crucial role in ensuring proper folding, stability, and accumulation (Pereira et al., 2014).

This study focuses on β-Casein fused to C-term Oleosin expression in Arabidopsis plant seeds. We tested different signal peptide sequences to endoplasmic-reticulum, vacuole, or chloroplast sub-cellular compartments, under the control of Glycinin seed specific promoter and terminator.

## Results

### Sub-cellular localization of target recombinant proteins in transgenic seeds

**Figure 1:**
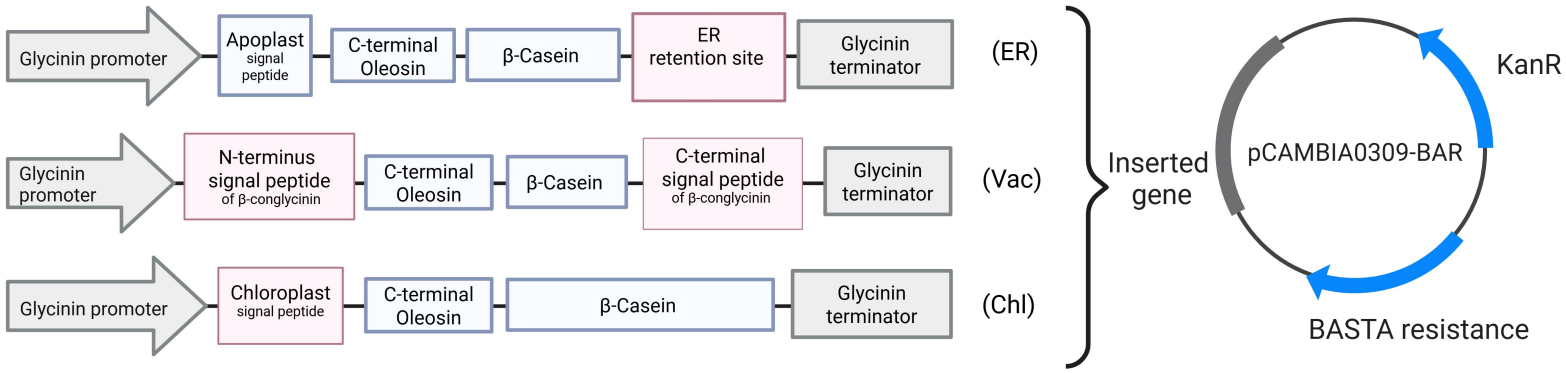
Schematic maps of the vector constructs used for Arabidopsis seeds genetic transformation, and the plasmid map (pCAMBIA 0309-BAR). (ER), endoplasmic reticulum; (Vac), vacuole; (Chl), chloroplast). Made with Bio Render.

Independent Arabidopsis transformants were generated using the floral dip method, with vectors designed to target the protein accumulation to specific subcellular compartments (**Figure 1**). For the vacuole (Vac), chloroplast (Chl), and endoplasmic reticulum (ER); 17, 23, and 19 seed lines were respectively harvested and characterized as detailed in the following sections.

### Expression of target recombinant proteins in transgenic seeds

**Figure 2:**
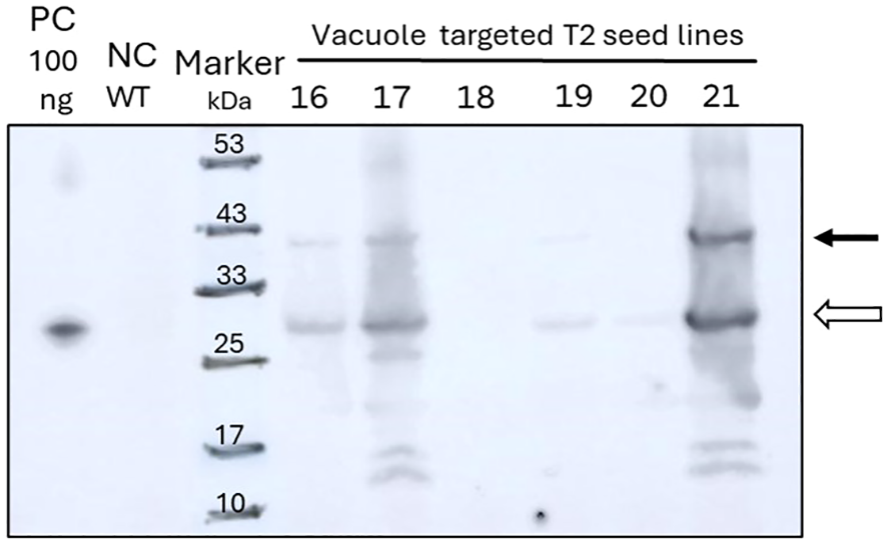
Western blot analysis of the vacuole-targeted recombinant protein expression in T2 transgenic Arabidopsis seed lysates. The solid arrowheads indicate the expected 42 kDa band of CTO-Cas, while open arrowheads indicate β-Casein devoid of the C-terminal Oleosin tag. Bovine β-Casein was used as a positive control (PC), and wild-type (WT) seed lysate served as a negative control (NC).

T2 independent seed lines were tested using a Western Blot analysis. Recombinant CTO-Cas (42 kDa) was detected in the seeds of some constructs (lines 16, 17, 19, 20 and 21 of the vacuole accumulation targeted plants). Furthermore, lower molecular weight bands were also observed, thought to indicate degradation products, among them a major band which correspond to β-Casein devoid of the C-term Oleosin tag (corresponding to the positive control) (**Figure 2**).

### Recombinant β-casein estimated quantities in transgenic mature seed lines

The accumulation of recombinant protein in selected T2 transgenic seed lines was evaluated by estimating its concentration per seed weight and as a fraction of the total soluble protein (TSP) extracted from the seeds. The quantification was performed by comparing the band intensity from Western blot analysis of seed lysates to that of a bovine β-Casein control protein, thereby providing a quantitative measure of recombinant protein expression across the transgenic lines.

**Table 1** summarizes the seed characteristics of vacuole targeted Line 21 compared to WT. The transgenic plant showed reduced seed yield (246 mg) and TSP content (24.2%) compared to WT (514 mg and 31.7%, respectively). Recombinant β-Casein accounted for 1.26% of the TSP.

**Table 1:** Accumulation of recombinant protein in T2 seeds transgenic lines, as W\W, and percentage from TSP seed extract..

			β-Casein accumulation	
Seed identity	Single plant seeds weight (mg)	TSP extracted from seeds (%)	from TSP (%)	from seed weight (ng/mg)
Wild Type	514 ±100*	31.7	n.a.	n.a.
Vacuole line 21	246	24.2	1.26	3669 **

*Data are seed weight means ± STDEV.P of twelve Wild Type independent plant lines in the same experimental settings.

**Indicates that the accumulation levels, calculated from bands of supernatants and pellets.

### Germination tests for transformant lines

**Figure 3:**
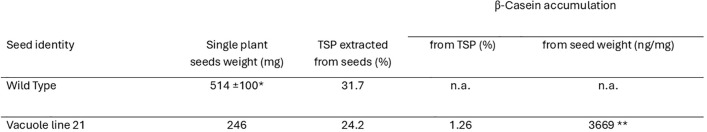
Accumulation of recombinant protein in T2 seeds transgenic lines, as W\W, and percentage from TSP seed extract. *Data are seed weight means ± STDEV.P of twelve Wild Type independent plant lines in the same experimental settings. **Indicates that the accumulation levels, calculated from bands of supernatants and pellets.

The selected lines expressing the target protein were evaluated for their germination capabilities in comparison to the WT. (**Figure 3**) Small portions of the seeds were cultivated on half-strength MS medium in triplicate Petri plates within a walk-in growth chamber, and germination rates were assessed after seven days. Among the lines tested against the WT threshold germination rate of 83%, certain lines exhibited lower germination percentages while line 21, also expressing the highest level of β-Casein, exhibited excellent germination rate, recorded at 91%.

### Transmission electron micrographs of pre immunogold-labeled β-casein in seed cell sections

**Figure 4:**
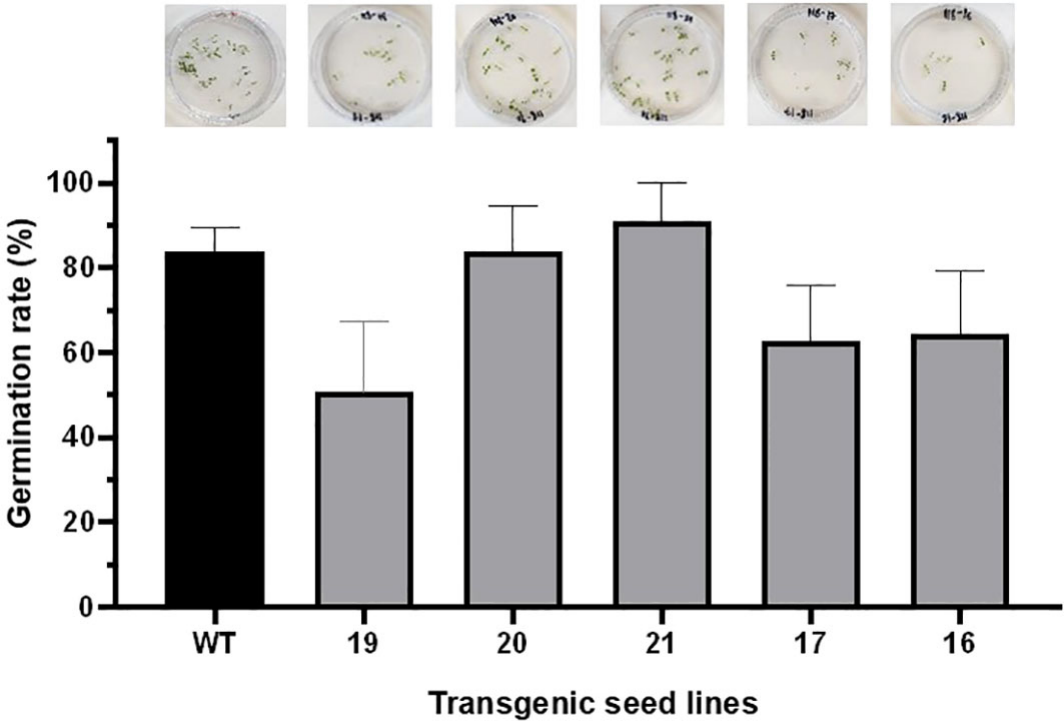
Germination rates (%) of Arabidopsis transgenic seed lines compared to WT, black column. Vac, line of vacuole targeted construct. TSP, Total Soluble Protein; n.a., data not available.

Immunolocalization of the expressed recombinant β-Casein in seed cell sections, was viewed using Transmission Electron Microscopy (TEM). To determine the protein subcellular localization, immune staining with polyclonal Bovine β-Casein antibody (Biomatik: 31876) were conducted. First, significant morphological changes were observed in the cellular compartments of the transformant lines compared to the WT control, as shown in **Figure 4B** and in **Figure 4A**, respectively. Specifically, the oil-bodies structure appeared to be disrupted, with the recombinant protein forming distinct clusters within the cell center.

**Figure 5:**
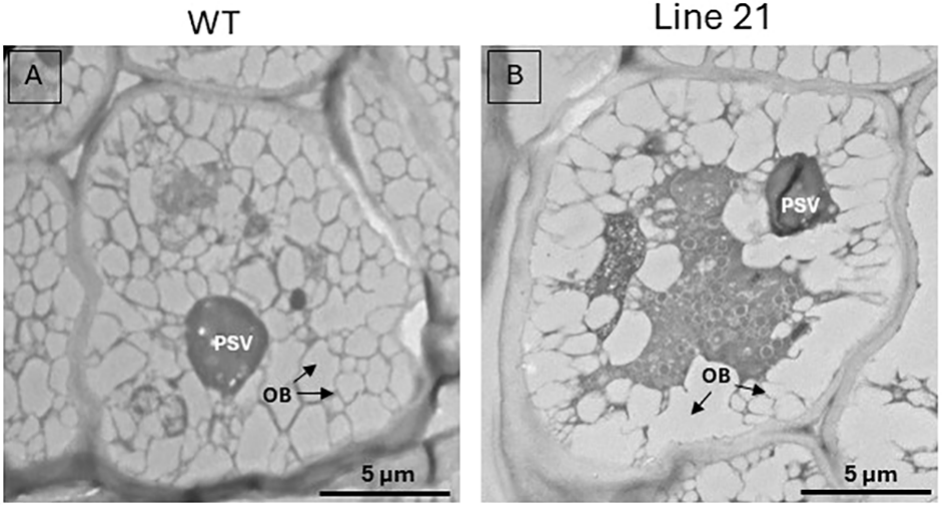
Morphologic view of arabidopsis whole seed cell section. **(A)** WT **(B)** Transgenic Line 21. (Scale bar = 5 μm). PSV, protein storage vacuole, OB, oil bodies.

**Figure 6:**
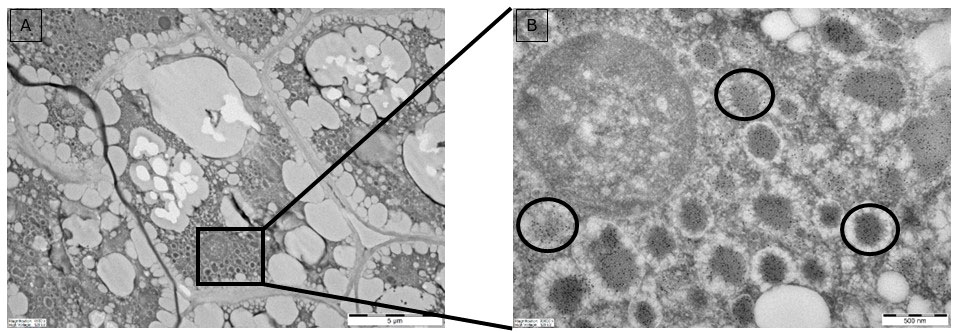
Immunolocalization by 12 nm gold nanoparticles of b-Casein in Line 21 seed cell sections using TEM, black circles represent protein clusters. The black square in **(A)** represents the area magnified in **(B)**. Scale bars: **(A)** 5 μm; **(B)** 500 nm.

Double immunogold labelling was performed on Line 21 seed cell sections to determine whether the electron-dense protein aggregates observed in **Figure 5**, represent protein storage vacuoles (PSVs) or pre-vacuole bodies. While the same with polyclonal Bovine β-Casein antibody (Biomatik: 31876) was used to observe the recombinant protein, an anti-12S seed storage protein (SSP) antibody (Agrisera: AS204403), known to specifically localize to vacuole compartment, was used to visualize the protein storage vacuoles (PSVs). 6 nm and 12 nm gold-conjugated nanoparticles were used to label PSVs and β-Casein, respectively. While the majority of protein aggregates were positively marked by 12 nm gold particles, indicating the presence of the β-Casein, these aggregates did not present labeling by the 6nm gold particles, indicating that these aggregates are not PSVs. This is supported by the separate labeling of PSVs with 6 nm gold nanoparticles, which clearly distinguished PSVs from the aggregates (**Figure 6**).

To definitively distinguish between the two immunogold signals within electron-dense regions, a quantitative particle distribution analysis was performed. In the structural PSV compartment, the signal was predominantly populated by the 6 nm particles (SSP marker), which accounted for 92.7% of the total counted particles (480 out of 518 particles), with minimal cross-labeling from the 12 nm particles (7.3%). Conversely, within the distinct target-protein-associated aggregates, the distribution was heavily inverted, with the 12 nm gold particles forming most of the signal. This strong spatial segregation suggests that the target-protein-containing structures represent independent, oil-body-associated aggregates rather than abnormal, PSV-derived compartments.

**Figure 7:**
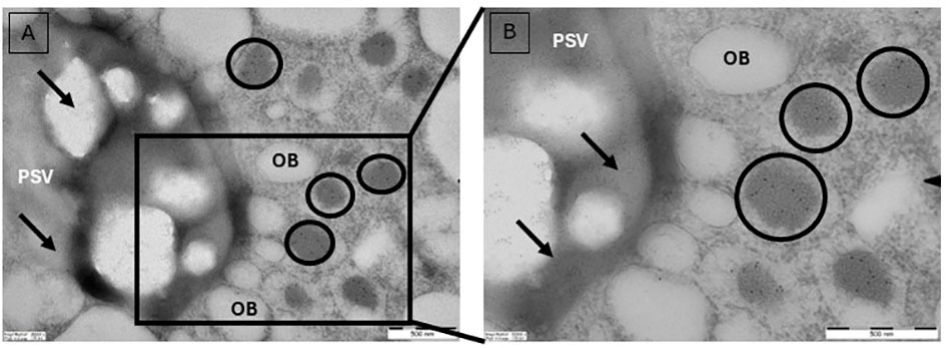
Immunolocalization of the native 12S seed storage protein (SSP) and b-Casein in Line 21 seed cells using 6 nm and 12 nm gold nanoparticles, respectively, visualized by transmission electron microscopy (TEM). 6 nm gold particles (black arrows) specifically mark protein storage vacuoles (PSVs), while 12 nm gold particles (black circles) indicate the presence of the targeted protein, b-Casein. The black square in **(A)** represents the area magnified in **(B)**. Scale bar = 500 nm. PSV, protein storage vacuole; OB, oil body.

Furthermore, large oil bodies dominated WT seed cells (**Figure 4A**), while seeds expressing CTO-Cas targeted to the vacuole had significant fewer large oil bodies, many more small oil bodies populated, those present often displaying aberrant coalescence with dense sub-cellular bodies only observed in the transgenic seed, which were positively gold-immunolabeled with anti-Casein antibodies (**Figure 5**) as well as anti-oil Body Associated Protein (OBAP1) (UniProt= Q9ZVY7) antibodies (PhytoAB: PHY0771A) (**Figure 7**), indicating close association of CTO-Cas and small oil bodies.

## Discussion

The applicability of plant systems for large-scale production of recombinant dairy products critically depends on achieving high protein yields. A promising approach to increase the accumulation of recombinant protein in plant seeds is to select a suitable plant intracellular compartment (Ma et al., 2003; Wei et al., 2004; Pereira et al., 2014; Solymosi and Schoefs, 2019; Kaushik et al., 2022; Morandini et al., 2011). Here, the chimeric protein (β-Casein fused to C-terminal oleosin) was successfully detected at approximately 42 kDa in mature vacuole-targeted transgenic Arabidopsis seeds (**Figure 2**). In contrast, no accumulation was observed in chloroplast- or endoplasmic reticulum targeted lines. These targeting events and the resulting morphological changes inherently take place during active seed maturation, when cellular transport systems are dynamically engaged in storage deposition. This distinct difference underscores the critical role that correct subcellular targeting plays in recombinant protein stability during seed development, aligning with previous observations (Takaiwa, 2013; Soledad et al., 2016; Rozov and Deineko, 2022; Muthusamy et al., 2024).

Among many possible explanations, rapid degradation, mis-localization caused by C-terminal oleosin topology and import failures within the endomembrane plant cell transportation system may explain the lack of accumulation in the ER or chloroplast compartments,as discussed by Hüttner and Stresser 2012, showing the ER-associated degradation (ERAD) pathway and the unfolded protein response (UPR) are essential mechanisms in plants for maintaining protein homeostasis. The synthesis of recombinant proteins under the control of an intensive promoter, such as Glycinin in transgenic seeds, can exceed the endoplasmic reticulum (ER) folding capacity, leading to ER stress. In response, the Unfolded Protein Response (UPR) is activated to restore cellular balance by targeting and degrading misfolded or unfolded proteins, thereby alleviating ER stress (Hüttner and Strasser, 2012). This may be particularly relevant to β-Casein, which is intrinsically unstructured phosphoprotein that dictates its functional properties and lack of stable secondary elements, making it inherently prone to proteolysis (Huppertz et al., 2017). While the ER is a productive compartment for recombinant protein expression, its quality control systems are designed to identify proteins that do not reach a stable, folded state. It is possible that the lack of a defined tertiary structure in β-Casein leads to its recognition as an unfolded polypeptide. Consequently, instead of accumulating, the protein might be targeted for degradation via the ERAD pathway. Such a mechanism could suggest why β-Casein levels remain beneath the detection threshold.Though successfully expressed, protein cleavage was observed across a few vacuole-targeted independent seed lines, specifically lines 16, 17, and 21 (**Figure 2**). A repeated major band equivalent to the native Bovine control protein was observed. A possible explanation to this phenomenon may be due to the amino acid substitutions at five phosphorylation sites in the β-Casein sequence, using negatively charged amino acids such as glutamic acid (D) or aspartic acid (E) to alter the isoelectric charge and achieve improved functional properties, i.e., “Phosphomimicking”, or simply the fusion junction between the two proteins, may have generated new unintended recognition sites with high affinity to native Arabidopsis seed cell proteases in the cytoplasm or specific compartments, which may explain the undesired cleavage (Bijl et al., 2014; Acevedo Fani and Abramson, 2023).

While oleosin-based expression systems are traditionally recognized for their ease of oil-body purification via flotation centrifugation, this strategy was intentionally bypassed in the current study. Due to the structural disruption and coalescence of the oil bodies observed in the vacuole-targeted line 21, standard flotation protocols would likely result in poor recovery, as the destabilized oil-protein aggregates could phase-separate unpredictably or pellet with cellular debris. Therefore, total protein extraction under denaturing conditions was utilized to ensure an accurate, quantitative assessment of the total accumulated recombinant protein within the seed tissue, preventing any artifactual loss during organelle isolation.

Nonetheless, Line 21 accumulated 1.26% chimeric protein of the total soluble protein (**Table 1**). These yields are competitive compared to previous studies; Maughan et al., 1999 reported bovine β-Casein expression levels of 0.1-0.4% of TSP in Glycine max (Soybean) seeds. Another study, conducted by Sovyanhadi et al., 2001, reported the expression of approximately 0.01–0.05% human β-Casein of TSP in Solanum Lycopersicum L. (Tomato) leaves. In terms of protein recovery, dry grinding of the seeds followed by alkaline buffer extraction employed in this study achieved 20-40% recovery of total soluble protein from seed lysates (**Table 1**). This percentage reflects efficient extraction and is considered typical for Arabidopsis seed protein recovery, given that the protein content in mature seeds of wild type (WT) variety is reported to be 30.5% (Demeyer et al., 2012). The average seeds yield per transformant plant of selected expressing lines (n=6), were 250 ± 110 mg, which presents lower yields compared to the Wild Type seed lines (n=12), of 500 ± 100. While transformant seed lines that expressed the chimeric protein were tested *in-vitro* for their germination ability (**Figure 3**), no correlation was found between protein expression levels and germination rates across the tested lines.

For example, line 17 exhibited medium protein expression with low germination rates, while line 20 showed low expression but high germination. Notably, the highest β-Casein accumulating line 21 reached 91% germination, exhibited excellent germination rate, suggesting that high expression levels did not necessarily impair seed viability (**Figures 2**, **3**). These findings are consistent with previous reports regarding recombinant protein expression in seeds (Arcalis et al., 2019; Kanai et al., 2023; Matzke and Matzke, 1998; Song et al., 2018).

Arabidopsis seed sections of Line 21 were immunogold-labelled for the detection of chimeric β-Casein by Transmission Electron Microscopy (TEM). In plants, seeds offer specialized organelles for protein storage, including Protein Storage vacuoles (PSVs) (Galili and Herman, 1997; Herman and Larkins, 1999; Park et al., 2004). In this study, aggregates containing a high concentration of 12n nm gold nanoparticles labeled β-Casein were observed, as presented in **Figures 5**, **6**. The observed protein aggregates in **Figure 5** are distinct in appearance from normal PSVs as shown in **Figure 4A**. Subsequently, as shown the double-staining experiment presented in **Figure 6**, although the chimeric protein is expressed at relatively high concentrations (**Figure 2** , **Table 1**) and directed for expression in the Protein Storage vacuole compartment (**Figure 1**), the spherical protein aggregates do not seem to correlate with vacuoles.

**Figure 8:**
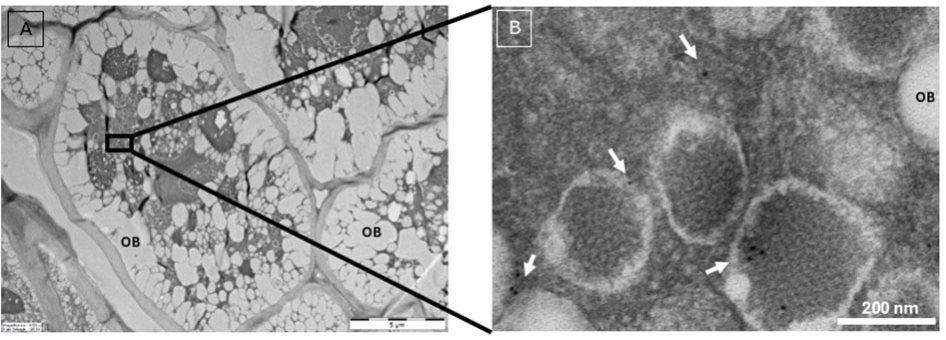
Immunolocalization of native OBAP1 in Line 21 using 12 nm gold particles, visualized by transmission electron microscopy (TEM). 12 nm gold particles (white arrows) specifically mark OBAP1. The black square in **(A)** represents the area magnified in **(B)**. Scale bars: **(A)** 5 μm; **(B)** 200 nm. Show positive staining of OBAP1 with 12 nm gold particles. OB, oil body.

However, the light ‘halo’ surrounding the labeled chimeric protein aggregates, which resemble those of oil bodies, could potentially represent oil bodies containing anchored C-terminal Oleosins fused to β-Casein (CTO-Cas) on their surfaces (**Figures 5**, **6**). While oil bodies in WT Arabidopsis seeds typically exhibit diameters ranging from 500-1,500 nm, the aggregates in the transgenic lines were characterized by slightly smaller dimensions, between 300–600 nm. Seed sections of Line 21 were immunogold-labelled again, this time for the detection of OBAP1 by Transmission Electron Microscopy (TEM). López-Ribera et al., 2014 suggest that OBAP1 protein is involved in oil body stability; therefore, its localization serves as a marker for the presence of oil bodies. **Figure 8** shows positive detection of the OBAP1, with 12nm gold particles localized at the periphery of the spherical structures, consistent with observations in Rapeseed López-Ribera et al., 2014. Combining this evidence with the strong labaling of β-Casein in the same spherical structures (**Figure 4**), strongly suggests that these structures represent oil-body-associated aggregates. This observation suggests a potential for efficient binding of Oleosin fusions to oil bodies, potentially enhancing their effectiveness for recombinant protein production (Bhatla et al., 2010; Yang et al., 2015; Zhai et al., 2018).

It is further observed that the accumulation of recombinant proteins in seeds can significantly alter the arrangement of endogenous oil bodies (OBs). In the transgenic Line 21, Transmission Electron Microscopy (TEM) analysis revealed two distinct populations of OBs: small, electron-dense structures and larger, irregularly shaped OBs exhibiting aberrant morphology (**Figure 4B**). The prevalence of smaller OBs following the expression of oleosin-fused proteins is consistent with previous reports, such as the production of human epidermal growth factor (hEGF) in Arabidopsis seeds, which similarly resulted in reduced OB size (Qiang et al., 2020).

**Figure 9:**
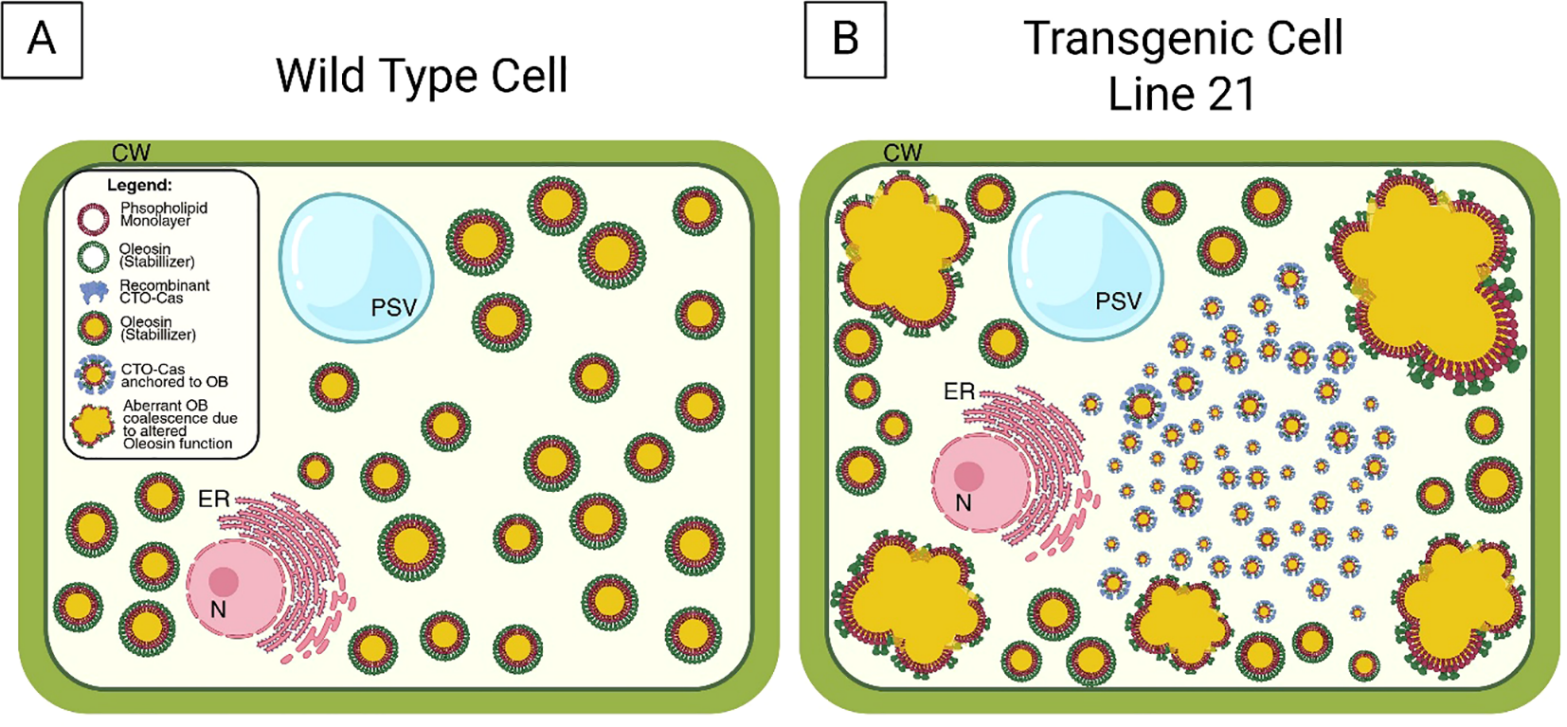
Schematic model of typical seed cotyledon storage cell depicting the morphology of aberrant oil body coalescence in transgenic seeds compared to Wild-Type. **(A)** Wild-Type: Schematic illustration of normal oil body (OB) architecture. OBs are characterized by a stable phospholipid monolayer coated with a balanced distribution of native oleosin proteins. **(B)** Transgenic Line 21 (vacuole-targeted): The central cellular region is populated by small, uniform oil bodies with recombinant CTO-Cas anchored to the outer phospholipid monolayer. In contrast, the cell periphery exhibits large, fused, and distorted OB’s. This aberrant morphology results from altered oleosin activity, which disrupts the stability of the phospholipid monolayer and drives uncontrolled coalescence. CW, cell-wall; ER; endoplasmic reticulum; PSV, protein storage vacuole; N, Nucleus; CTO-Cas, C-terminal Oleosin fused to Bovine b-Casein. Made with Bio Render.

Taken together with the immunogold labeling results from **Figures 5**, **8**, these morphological shifts further support the notion of an association between the CTO-Cas chimeric protein and the oil body membrane. Native Oleosin is the primary structural protein responsible for maintaining the stability and characteristic spherical shape of endogenous oil bodies (Bhatla et al., 2010). This essential stabilizing activity appears to be compromised by the presence of the C-terminal oleosin fused to the recombinant β-casein, which increases the affinity of the CTO-Cas to the lipid surface. The simultaneous presence of both native and chimeric oleosins likely creates a competitive environment on the oil body (OB) membrane. While the oleosin moiety facilitates the anchoring of the recombinant cargo, its “over-utilization” or redirection within the CTO-Cas fusion may interfere with the natural ontogeny or coalescence of the OBs. This competition may lead to the distorted phenotype and aberrant coalescence, as illustrated in our proposed model in **Figure 9**.

## Experimental procedures

### Plant material and growth conditions

Arabidopsis (A. Thaliana) wild-type Col-0 ecotype plants were used in this work. Seeds were sown in sterilized Even Ari 761 soil mixture (Even Ari Green), combined with Osmocote^®^, a 6-month slow-release fertilizer (Sierra Chemical Co.), in a ratio of 16 gr fertilizer per 4 li of soil. Seeds were kept at 4 °C for 3 days in the dark for stratification and then transferred to standard growth conditions, as detailed below. Plants were growth during 2024–2025 in the controlled green house facility at The Robert H. Smith Institute of Plant Sciences and Genetics, Faculty of Agriculture, Food and Environment, The Hebrew University of Jerusalem [31.904203, 34.80190]. The greenhouse temperatures were kept at 22 °C with natural daylight for 16 hours, and 19 °C by night for 8 hours. To provide a long day photoperiod regime, supplemental evening and morning light was provided in the greenhouse during winter, and shade cloth regulating the light intensity and temperature in the summer. An agrotechnical treatment throughout the plant life cycle was carried out according to The Arabidopsis Information Resource (TAIR protocol).

### Vector construction and plant transformation

Three types of chimeric genes were constructed in binary plasmid Pcambia0390-BAR, which contain Glycinin specific seed promoter and terminator (UniProt P04776).The cassette contained the protein sequence of Bos taurus (Bovine) β-Casein (UniProt P02666) with five amino acid substitutions at the estimated phosphorylation sites, as described in the patent “Modified dairy proteins, methods for their production and use” (Acevedo Fani and Abramson, 2023). Specifically, the BC3 (β-Casein No. 3) sequence was modified at its five phosphorylation sites by substituting the serine residues with negatively charged amino acids, such as glutamic acid (D) or aspartic acid (E), at positions 15 (S15→D), 17 (S17→E), 18 (S18→D), 19 (S19→E), and 35 (S35→D) within the 209-amino-acid full-length sequence. These substitutions were designed to alter the isoelectric charge. This sequence was fused to the Arabidopsis thaliana (mouse-ear cress) Oleosin protein sequence (118-173) (UniProt P29525), specifically the C-terminus Oleosin, as described in patent “C-term Oleosin” (matter code: P10199WO). This fusion protein was attached to 3 different signal peptides sequences, targeting the protein to endoplasmic reticulum (ER), chloroplast (Chl) and vacuole (Vac). The ER cassette contained an Apoplast signal (Q9CAC1) from AtCel1 protein (A. Thaliana), that was inserted to the N-terminal site of the chimeric protein sequence (Shani et al., 2006). In addition to the AtCel1 signal peptide, an ER-retention motif KDEL was fused to the C-terminus of the protein, ensuring the protein retention within the ER (Denecke et al., 1992). The vacuole cassette carried a dual targeting mechanism, involving both the first 23 amino acids of the N-terminal propeptide and the C-terminal signal peptide of the β-conglycinin (F7J077) from Glycine Max (Soybean) (Nishizawa et al., 2006). In the chloroplast cassette, we harnessed the transit peptide of potato rbcS protein (P26574) from Solanum tuberosum (Wolter et al., 1988) Schematic presentation of the different constructs presented in **Figure 1**. Codon optimization for gene cassettes and plasmids synthesis was outsourced and conducted by Twist Bioscience (location). These vectors were introduced into A. Tumefaciens (Gv3103) for the transformation. Agrobacterium-mediated transformation of Arabidopsis was performed using the floral-dip method as described previously (Clough and Bent, 1998).

### Transformant seed line selection, weight and germination

The expression vector was designed using a pCambia0309 vector with a phosphinothricin acetyltransferase gene (blpR), conferring resistance to BASTA in stably inherited transgenic plants. Seeds of the putative primary (T1) transgenic plants were grown on soil parallel to wild type seeds. Selection of 100 mg floral dipped seeds was conducted on-soil, with 3 sequential applications of 30 mg/li glufosinate ammonium (BASTA) in an interval of 4 days starting from the two-leaf stage. This procedure identified independent T2 transformant plants among T1 seed bulks (Hadi et al., 2002). To evaluate sufficient number of independent lines, the procedure was conducted multiple times. Plants were cultivated simultaneously under consistent conditions, and seeds were harvested upon plant maturity. Seeds were gathered from at least 15 independent lines for each, weighed and stored. Standard plant germination tests were conducted on half-strength Murashige and Skoog (MS) medium supplemented with 1% (w/v) sucrose to assess general seed viability, utilizing separate aliquot of seeds from the same harvested lines used for Western Blot and TEM analyses. Seeds were kept at 4 °C for 3 days in the dark for stratification, then transferred to normal growth conditions. The Petri plates were set in a walk-in growth chamber kept at 22 °C with a light intensity of 80 μmol m−2 s−1 and a 16-h light/8-h dark photoperiod (Murashige and Skoog, 1962).

### Protein extraction from seeds and western blot analysis

Seed lines (T2) in tubes containing metal beads were pre-chilled by submerging in liquid nitrogen, and dry ground four times for 30 seconds at a shake frequency of 30/second using a TissueLyser II (QIAGEN). The powdered tissue was then mixed with sample application buffer at a ratio of 1 mg of seeds to 60 μl SABx1, followed by incubation at 95 °C for 20 minutes. Subsequently, (Bovine) β-Casein (SIGMA C6905), 10 μl of the transgenic seed lysate samples and WT were loaded onto a 4-20% gradient SDS-PAGE gel, run for 35 minutes at 200V, and then was transferred to the membrane. The membrane was blocked for 1 hour at room temperature with 5% (w/v) Bovine Serum Albumin (SIGMA A7030) buffer and then incubated overnight at 4 °C with Polyclonal Bovine β-Casein antibody (Biomatik: 31876, 1:10000). The membrane was subsequently washed three times for 5 minutes each using Tris Buffer Saline with Tween 20 (TBST) and incubated again for one hour at room temperature with Goat Anti-Rabbit antibody (Goat Anti-Rabbit IgG Antibody, HRP-conjugate, 12-348, Millipore, 1:10000). The membrane was washed three more times for 5 minutes each using TBST and developed using Clear band ECL, a Western blot substrate kit.

### Quantification of recombinant β-casein from transformant seeds

Positive (T2) seed lines were dry ground as described in the last paragraph. The powdered tissue was then mixed with an alkaline buffer, specifically a Carbonate-Bicarbonate buffer (pH=10.6). The total protein content of the supernatant was determined using the bicinchoninic acid (BCA) protein assay (Micro BCA Protein Assay Kit, 23235, Thermo Scientific), following the manufacturer’s instructions. The total soluble protein (TSP) concentration (ug/ml) was then converted to TSP percentage based on the ground seeds and the added buffer volume. Additionally, the TSP (ug/ml) of each transformant line was calculated to load a normalized amount of 20 mg TSP from all transformant lines onto a protein electrophoresis gel. Bovine Serum Albumin (Sigma A7030) at four different concentrations served as reference points. The membrane was blocked for 1 hour at room temperature with 5% (w/v) Bovine Serum Albumin buffer and then incubated overnight at 4 °C with Polyclonal Bovine β-Casein antibody (Biomatik: 31876, 1:10000). The membrane was subsequently washed three times for 5 minutes each using Tris Buffer Saline with Tween 20 (TBST) and incubated again for one hour at room temperature with Goat Anti-Rabbit antibody (Goat Anti-Rabbit IgG Antibody, HRP-conjugate, 12-348, Millipore, 1:10000). The membrane was washed three more times for 5 minutes each using TBST and developed using Clear band ECL, a Western blot substrate kit. The band quantification assay, conducted according to the Image Quant™ TL (Cytiva) analysis software procedure, the band of 100 ng BSA determined as the final reference point.

### Post-embedding immunogold labeling of LRW sections

Dry Arabidopsis WT and 2 seed lines which positively accumulate the chimeric protein, were placed on adhesive tape and a small portion of the seed was cut out with a sharp knife, in order to allow processing materials easy access to the embryo. The seeds were fixed in 2.5% glutaraldehyde, 0.1% paraformaldehyde, in 0.1 M cacodylate buffer (pH7.4) for 1 hour at 4 degrees. The seeds were rinsed 4 times in cacodylate buffer and dehydrated in increasing concentrations of ethanol consisting of 50%, 70% 80% and 90% ethanol. Following dehydration, the seeds were infiltrated with gradual LRW concentrations, starting with 90% ethanol and LRW at a 1:2 ratio, and two changes of 100% LR White at room temperature. Then 100% LR White at 40 °C overnight. After 2 more changes of 100% LR white at room temperature, the tissue was embedded in fresh resin and let polymerize in an oven at 50 °C for 24 hours. Embedded tissues in blocks were sectioned with a diamond knife on Leica Reichert Ultracut S microtome and ultrathin sections (80nm) were collected onto 200 Mesh, thin bar coated carbon-formvar nickel grids. For immunolabeling grids were incubated in drops of different reagents on parafilm within a wet chamber: LRW sections were etched in saturated aqueous sodium metaperiodate solution, followed by immersion in 1% sodium borohydride. Sections were washed 5 times in a working solution made of Tris-buffered saline (TBS) containing 20 mM Tris-base, 0.9% NaCl, 0.5% BSA, 0.5% Tween-20 and 0.13% NaN3, pH 8.2. This was followed by 30 minutes incubation in blocker containing 5% normal goat serum (NGS) in the working solution. Sections were exposed to Polyclonal Bovine β-Casein antibody (Biomatik: 31876 or 32521), or Polyclonal 12S seed storage protein CRC antibody (Agrisera: AS204403) at room temperature and then moved to 40C for overnight incubation. The antibodies were diluted 1:50 in 1% goat serum in the working buffer. For immunogold labeling we used 12nm colloidal gold Goat anti Rabbit IgG (Jackson ImmunoResearch Laboratories). This incubation was followed by fixation of 2% glutaraldehyde and contrasting in saturated aqueous uranyl acetate and lead citrate before air-drying. The grids were viewed with Tecnai 12 TEM 120kV (Phillips, Eindhoven, the Netherlands) equipped with Phurona camera and RADIUS software (Emsis GmbH, Münster, Germany). Quantitative immunogold particle analysis and distribution counts were performed on representative micrographs using ImageJ software (version 1.54, NIH, USA).

## Data Availability

The raw data supporting the conclusions of this article will be made available by the authors, without undue reservation.
